# Sustained abstinence in severe ketamine use disorder following ibogaine treatment case report

**DOI:** 10.3389/fpsyt.2026.1846320

**Published:** 2026-07-20

**Authors:** Sergio R. Pérez Rosal, Sonya C. Faber, Markus Backmund

**Affiliations:** 1 Medizinische Hochschule Brandenburg Theodor Fontane, Neuruppin, Germany; 2 Psychedelia-Stiftung, Berlin, Germany; 3 School of Psychology, University of Ottawa, Ottawa, ON, Canada; 4 P3 Akutklinik für Psychiatrie, Psychotherapie und Psychosomatik, Tutzing, Germany; 5 Ludwig-Maximilians-Universität (LMU) Munich, Munich, Germany

**Keywords:** case report, ibogaine, ketamine use disorder (KUD), psychoactive substances, substance use disorder (SUD)

## Abstract

Substance use disorders (SUD) involving ketamine, cocaine, and alcohol present significant clinical challenges, often characterized by high relapse rates and limited pharmacological options. This case report details a 30-year-old male with a five-year history of severe polysubstance dependence, including daily intranasal ketamine use (2–3 g/day), cocaine, and alcohol, comorbid with recurrent depressive disorder. Despite conventional psychiatric treatment, the patient experienced severe cravings and sought ibogaine-assisted treatment. The patient underwent a structured 13-day residential program in Mexico, receiving an 800 mg ibogaine HCl flood dose (10.1 mg/kg), followed by two supplementary booster doses of 300 mg (3.8 mg/kg) under continuous medical and ECG monitoring. Following treatment, the patient reported an immediate cessation of cravings for all substances. Over approximately 17 months of follow-up including a medically supervised fractionated ibogaine intervention approximately 11 months after the initial treatment, serial toxicology and psychometric assessments were consistent with continued abstinence from ketamine, cocaine, alcohol and other previously misused substances, and significant improvements in depression (PHQ-9: 0–3), anxiety, and quality of life (WHOQOL-BREF: 55 to 71). This report represents the first longitudinally documented case of sustained abstinence in severe ketamine use disorder following ibogaine treatment, supported by serial toxicology and standardized psychometric outcomes. It adds objective, time-resolved evidence to a literature that has largely focused on ibogaine for opioid use disorder, and it highlights ketamine use disorder as a specific target for future controlled trials. The therapeutic outcome is hypothesized to arise from ibogaine’s unique polypharmacology. This includes acute NMDA antagonism, which may disrupt compulsive circuits, as well as noribogaine’s (the principal long-acting metabolite of ibogaine, with an elimination half-life of approximately 28–49 hours) kappa opioid receptor agonism and serotonin transporter inhibition, which stabilize reward pathways. Additionally, enhanced neuroplasticity via GDNF/BDNF expression may facilitate long-term behavioral changes. This case provides rare, rigorously documented evidence for ibogaine’s potential in treating chronic ketamine dependence and highlights the urgent need for controlled clinical trials within regulated frameworks to further investigate its safety and efficacy.

## Introduction

Ketamine, cocaine, and alcohol use disorders have become an increasing challenge for clinicians, particularly among young adults with psychiatric comorbidities. Despite intervention, reports of ketamine dependence are on the rise globally, characterized by cognitive toxicity and high relapse rates (1). This escalating public health challenge is underpinned by a distinct neurobiological substrate involving glutamatergic dysregulation; chronic NMDA receptor antagonism alters synaptic plasticity and paradoxically increases serum brain-derived neurotrophic factor (BDNF) levels (2). Clinically, frequent dissociative misuse in young adults carries severe psychiatric risks, demonstrating a strong positive correlation with psychotic-like experiences that complicate comorbid psychopathology (3). Cocaine disorders remain particularly difficult to treat, with no approved relapse-prevention pharmacotherapies and long-term abstinence rates below 20% in conventional treatment programs. Comorbid alcohol use and depression further degrade outcomes, as polysubstance use increases craving while reducing treatment retention.

Current strategies for treating stimulant and ketamine dependence rely primarily on psychosocial interventions, occasionally augmented by antidepressants, benzodiazepines, or off-label medications such as naltrexone, baclofen, or lamotrigine. Although ketamine-assisted therapy shows efficacy in treatment-resistant depression (4) and alcohol dependence (5), clinical evidence for treating chronic, compulsive ketamine addiction itself remains scarce.

### Ibogaine: pharmacology and controversy

**Figure 1:**
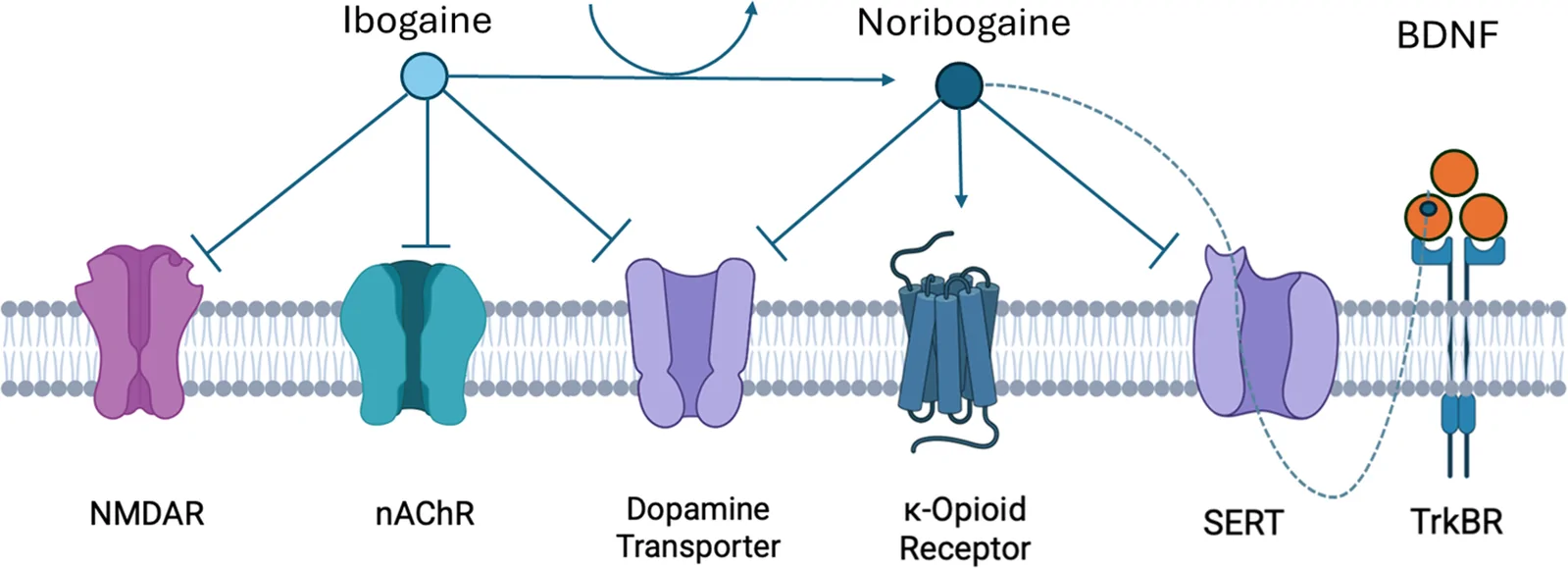
Major mechanisms of action for ibogaine. Major mechanisms of action for ibogaine & noribogaine. 1. Acute Dampening of the Reward Signal and Anti-Dysphoria: Ibogaine and its metabolite, Noribogaine, directly reduce the acute, high-amplitude release of dopamine (the primary reinforcement signal) triggered by drugs or associated cues, particularly via NMDA antagonism. This effect is complemented by Noribogaine’s critical kappa opioid receptor KOR agonism, which may contribute to the rapid interruption of withdrawal-related dysphoria and craving (4, 11). This collective action rapidly dampens the exaggerated reward signal and immediately weakens the compulsive drive for use. 2. Synaptic Stabilization and Withdrawal Mitigation: The longer-acting metabolite, Noribogaine, gently modulates the system. Unlike addictive stimulants, Noribogaine acts as a moderate blocker of the dopamine transporter DAT, which clears dopamine from the synapse. This helps maintain a stable, non-peaking level of background dopamine, which prevents the severe emotional crash and dysphoria of withdrawal while simultaneously avoiding the strong, new reward signals that drive addiction (12). 3. Disruption of Learned Associations: Ibogaine and Noribogaine chemically disrupt the learned associations that drive addiction. By strongly blocking receptors (specifically the α3β4 nAChR), they weaken the brain’s link between environmental cues (triggers) and the automatic, dopamine-driven habit. Individuals report that the triggers simply lose their power to provoke craving (13). 4. Induction of Neuroplasticity: Ibogaine induces a temporary surge in neuroplasticity (the brain’s ability to reorganize). By activating pathways such as those involving Brain-Derived Neurotrophic Factor BDNF and its receptor TRKB, it opens a critical window of opportunity where the brain can easily form new, healthier behavioral patterns to replace the previously weakened addiction habits (14, 15).

Ibogaine, an indole alkaloid derived from *Tabernanthe iboga*, has been used in West African ritual contexts for centuries and gained attention for its reported anti-addictive properties in the late 20th century (6). As a substance with a complex pharmacology, its primary anti-addictive effects are hypothesized to stem from non-competitive NMDA antagonism and α3β4 nicotinic acetylcholine modulation, which disrupt conditioned drug-seeking behavior. Additionally, ibogaine and noribogaine, the principal long-acting metabolite of ibogaine, with a plasma elimination half-life of approximately 28–49 hours and persistent biological activity over 5–7 days (7, 8), act as kappa-opioid agonists and sigma-2 ligands while inhibiting serotonin and dopamine transporters. These diverse actions converge to increase the expression of glial cell line-derived neurotrophic factor (GDNF) and brain-derived neurotrophic factor (BDNF) in mesolimbic circuits (9). This neuroplastic surge is believed to underpin the sustained reduction in craving (10, 11) across various substance use disorders. The key molecular targets and proposed anti-addictive pathways are summarized in **Figure 1**.

Open-label studies and observational data suggest a possible rapid interruption of withdrawal and craving in opioid, cocaine, and alcohol dependence, followed by sustained reductions in use when combined with psychosocial support (16, 17). However, ibogaine remains unapproved in Europe and North America due to safety risks, including QT-interval prolongation, torsades de pointes (18), and cerebellar neurotoxicity. Worldwide fatalities (5) often involve unregulated settings lacking ECG or electrolyte monitoring.

### Summary of ibogaine clinical evidence

The evidence for ibogaine efficacy includes retrospective surveys, observational cohorts, and limited clinical trials. These studies report reduced withdrawal and cravings over days to months (19), alongside improved depression, anxiety, and PTSD symptoms. Notable data include a study of U.S. veterans using ibogaine (20) as well as a small, randomized study in which ibogaine significantly reduced cocaine craving and relapse while sustained abstinence beyond several months is often described, findings rely largely on self-report, small samples and inconsistent environments. (5, 21).

Safety data from phase I and open-label trials indicate that low-dose ibogaine and noribogaine are well tolerated, with no serious adverse events in supervised open label settings. However, the few published placebo-controlled trials also show that noribogaine causes dose-related QTc prolongation without clear opioid-agonist effects (7). One treatment-related death occurred in a medical setting where monitoring was substandard. Taken together, despite the scarcity of large-scale controlled data, current findings suggest clinical efficacy and justify further evaluation under regulated conditions (5, 21).

### Dose and safety summary

Reported ibogaine doses vary from microdosing to high total amounts. While a starting dose of 0.87 mg/kg is suggested as safe, published studies range from 0.28 mg/kg to 55 mg/kg. Among 24 studies reviewed (21), two deaths occurred: one in an informal setting involving heroin and another in a medical setting with high dosing, poor monitoring, and continued antidepressant use. The literature includes at least 58 additional ibogaine associated emergencies or fatalities, often involving polysubstance use and cardiac arrhythmias. Rare psychiatric complications such as psychosis and mania exist. Some reports suggest repeated low dose regimens reduce withdrawal with fewer safety concerns, though controlled evidence is lacking.

### Gap in the literature

Although evidence supports ibogaine use for opioid and cocaine addiction (5, 21), it is virtually non-existent for chronic ketamine dependence, particularly involving cocaine and alcohol. Existing reports often lack the prospective, objective rigor required for definitive clinical cases. This report represents the first longitudinally documented case of sustained abstinence in severe ketamine use disorder following ibogaine treatment, providing the spectrum of read-outs documented herein. This case uniquely incorporates longitudinal standardized scales for craving, depression, anxiety, insomnia, trauma, and quality of life. By detailing care across both conventional psychiatric and ibogaine treatment settings, this report offers a robust perspective on complex polysubstance conditions.

### Aim of this case report

We describe an adult male with severe ketamine, cocaine, alcohol, and nicotine dependence who left inpatient psychiatric treatment in Germany for ibogaine-assisted therapy in Mexico. He returned to the German clinic drug-free, achieved complete craving cessation, and remained abstinent over approximately 17 months of follow-up, confirmed by toxicology, psychometrics, and clinical observation. This case explores potential mechanisms and ethical considerations for applying ibogaine in treatment resistant stimulant and ketamine use disorders.

## Case description

### Patient information

**Figure 2:**
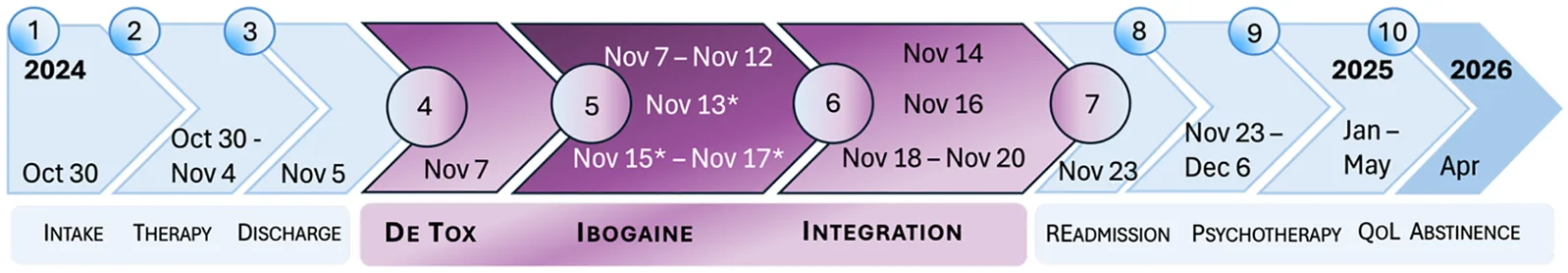
Patient journey. Longitudinal clinical trajectory and treatment timeline. The schematic illustrates the patient’s progression through ten distinct clinical stages: (1) initial presentation at a German psychiatric clinic with severe polysubstance use disorder (ketamine, cocaine, alcohol) and severe depression (BDI-II: 32); (2) receipt of supportive therapy and nutritional stabilization without antidepressants or opioid agonists; (3) discharge against medical advice to seek ibogaine therapy abroad; (4) intake at a 13-day residential program in Mexico including medical screening and a 7-day supervised detox; (5) administration of a 800 mg ibogaine HCl flood dose (10.1 mg/kg) under continuous ECG and 30-minute vital sign monitoring, followed by two booster doses of 300 mg each; (6) a post-dosing period focused on psychological processing and emotional regulation; (7) medical discharge following the reported complete cessation of cravings for all substances; (8) re-admission to the German clinic for integration therapy with abstinence confirmed via negative toxicology; (9) participation in daily psychotherapy and structured relapse prevention programs; and (10) eleven-month longitudinal monitoring via OutcomeMD confirming sustained abstinence, absent cravings, minimal depressive symptoms (PHQ-9: 0–3), and significant quality-of-life improvements (WHOQOL-BREF increase from 55 to 71).

A 30-year-old male with a 5-year history of ketamine, cocaine, and alcohol use disorder, alongside recurrent depression, voluntarily entered a German psychiatric clinic on 30 October 2024. He reported daily intranasal ketamine use (2 to 3 g/day; 10 to 15 g/week), frequent cocaine co-use (~1 g per session), episodic alcohol binges, and nicotine dependence. Symptoms included escalating cravings, depression, suicidal ideation without plan, insomnia, and functional decline. The patient had no history of psychosis, seizures, cardiovascular disease, liver or kidney disease, or prior ibogaine exposure (**Figure 2**).

On intake, mental status examination showed depressed mood, anhedonia, psychomotor retardation, and low self-worth, without psychotic features. Urine toxicology was positive for ketamine, cocaine, and benzodiazepines, but negative for alcohol. Baseline testing revealed a Beck Depression Inventory II (BDI II) score of 32 (severe) and globally elevated Symptom Checklist (SCL 90 R) values. While withdrawal was mild, craving was “unbearable.” During admission, he received psychotherapy, nutritional stabilization, melatonin (5 mg), B vitamins, and symptomatic benzodiazepines. No antidepressants or opioid agonists were initiated. On 5 November 2024, he left against medical advice to seek ibogaine detoxification. On 7 November 2024, he began a 13-day residential program in Mexico featuring medical screening, continuous monitoring, staged ibogaine dosing, and psychosocial support.

### Baseline assessment and medical eligibility

At intake, the patient showed severe substance use disorder with a Drug Use Disorders Identification Test (DUDIT) score of 34. Staff observed serial urine toxicology at admission, prior to the flood dose, and before each supplement; tests were consistently negative for ketamine, cocaine, alcohol, opioids, and stimulants, though benzodiazepine positivity was clinically expected. Withdrawal severity was low, with CIWA-Ar scores declining rapidly and remaining minimal. Laboratory testing at intake and surrounding the flood dose showed electrolytes, renal and hepatic function, and hematologic parameters within reference ranges. Multiple 12-lead ECGs at intake and prior to each dose showed sinus rhythm and appropriate QTc intervals for monitored ibogaine, confirming medical eligibility.

### Arrival, medical intake, and pre-treatment program

The patient arrived at the ibogaine center on Day 0. Medical intake on Day 1 included a 12-lead ECG, vitals, urine drug screening, and bloodwork for electrolytes, metabolic panel, and hepatic and renal function. No QTc prolongation, electrolyte abnormalities, or metabolic disturbances were found. Psychometrics showed severe substance use (DUDIT 34, AUDIT-C 3, CAGE-4). He reported last ketamine and cocaine use 24 to 36 hours prior. Baseline insomnia was moderate (ISI 15), while depression and anxiety were elevated (PHQ-9 18, GAD-7 15). Lifetime trauma exposure was screened using the Brief Trauma Questionnaire (BTQ; 22), a 10-item self-report measure of lifetime exposure to potentially traumatic events; the BTQ functions as an exposure screener rather than a symptom-severity instrument. Initial CIWA-Ar was 12; no alcohol or benzodiazepines were administered.

These measures excluded medical contraindications and established baseline status. A second 12-lead ECG on Day 7 confirmed cardiac stability before dosing. From Day 1 to 6, the patient completed a supervised 7-day detoxification with daily psychosocial support and coaching. No psychotropic medications were administered; melatonin was available on demand for sleep but was not part of the structured protocol.

### Ibogaine compound, dosing strategy

The center used encapsulated ibogaine-HCl. During the first admission, a staged protocol was used: a single 800-mg flood dose (10.1 mg/kg; body weight 79 kg) on Day 8, followed by two supplementary doses on Day 10 (300 mg; 3.8 mg/kg) and Day 12 (300 mg; 3.8 mg/kg). All doses were administered under continuous cardiac and vital-sign monitoring. Baseline QT/QTc was 372/395 ms; QTc-prolongation peaked at 541 ms before resolving below 500 ms within hours. The complete dosing schedule across the index treatment and the subsequent fractionated booster intervention is summarized in **Table 1**.

**Table 1:** Pharmacological mechanism.

Target/mechanism	Primary process affected	Ibogaine binding	Noribogaine binding	Mechanistic notes
[1] NMDA (PCP site)	↓ Phasic dopamine spikes	Moderate antagonist	Weak	Reduces VTA burst firing; interrupts drug-seeking (23)
[2] KOR	↓ dopamine responsiveness	Weak	Moderate	Critical anti-addictive and anti- dysphoric effects; KOR agonism is a preserved target in safer analogues (10, 23)
[3] DAT	↑ Tonic dopamine tone	Moderate	Moderate	Stabilizes dopamine baseline (24)
[4] α3β4 nAChR	↓ Phasic dopamine spikes	Strong antagonist	Weak	Weakens conditioned cue linkages; reduces reward & craving (10)
[5] σ-1 receptor	Plasticity modulation	Moderate	Weak	Possible TRKB facilitator
SERT	modulates mood and anxiety	Moderate	Strong inhibitor	Main serotonin transporter effect; reuptake blocker (24)
[6] TRKB (via BDNF)	↑ Neuroplasticity	Indirect	Indirect	Downstream convergent pathway (4, 14, 15)
NET	Minimal	Weak	Weak	Not central
5-HT2C	Minor modulation	Weak	Weak	Secondary
MOR	None	Very weak	Very weak	Not mechanistic (10, 23)
DOR	None	Very weak	Very weak	Not relevant (10, 23)
5-HT2A	Minimal psychotropic	Very weak	Very weak	Main target for serotonergic psychedelics (4, 25)

A systematic comparison of the known binding affinities and proposed functional consequences of ibogaine and its primary active metabolite, noribogaine, across central nervous system (CNS) receptors and transporters implicated in the pathophysiology of substance use disorders. The therapeutic efficacy of Ibogaine is hypothesized to arise from a biphasic mechanism: rapid and acute interruption of addiction circuits by Ibogaine (primarily via NMDA and nAChR antagonism) followed by prolonged, sustained anti-craving and neuroplastic effects mediated by Noribogaine (predominantly via KOR agonism and SERT inhibition). Data are compiled from in vitro binding assays and in vivo functional studies. N-methyl-D-aspartate (NMDA) receptor, α3β4 nicotinic Acetylcholine Receptor (α3β4 nAChR), Ventral Tegmental Area (VTA), Sigma-1 (sigma-1) receptor, Serotonin Transporter (SERT), Dopamine Transporter (DAT), Tropomyosin Receptor Kinase B (TRKB), Brain-Derived Neurotrophic Factor (BDNF), kappa-Opioid Receptor (KOR), Norepinephrine Transporter (NET), mu-Opioid Receptor (MOR), delta-Opioid Receptor (DOR), and 5-Hydroxytryptamine (5-HT2A/2C) receptor.

### Monitoring and safety procedures during ibogaine sessions

Ibogaine administration followed strict medical oversight. For the first 12 hours of the flood session, the patient was continuously monitored via 12-lead ECG, pulse oximetry, and blood pressure checks every 30 minutes. Prophylactic IV fluids were administered per the Beond cardiac-safety protocol: 0.9% saline 100 cc + 2 g MgSO_4_ over 20 minutes (07:50–08:10, pre-dose), followed by 0.9% saline 500 cc + 4 g MgSO_4_ + 20 mEq KCl over 4 hours (09:00–14:30), and 0.9% saline 500 cc over 6 hours (15:00–20:00), for total magnesium 6 g and total potassium chloride 20 mEq during the flood session. A licensed nurse remained present, with physicians and psychotherapists immediately available. The setting included eye masks and music to support an introspective experience. No serious cardiovascular events, seizures, or arrhythmias occurred. QTc values rose progressively from a pre-dose value of 395 ms, with hourly readings of 469 ms (Hour 1), 476 ms (Hour 2), 493 ms (Hour 3), and a peak of 541 ms at Hour 4 (12:00 noon, ~4 h post-administration), followed by spontaneous resolution: 524 ms at Hour 5, 504 ms at Hour 6, and 469 ms at Hour 7. No antiarrhythmic medication was administered at any point; the prolongation resolved spontaneously under the prophylactic magnesium/potassium regimen described above. Only mild, self-limited effects such as transient hypertension, ataxia, and emesis were observed. Hemodynamic stability was preserved, and no rescue interventions were required beyond routine supportive care.

### Post-session care and integration

Integration (Days 8, 10, and 12) focused on psychological processing and stabilization. Sessions facilitated by trained therapists addressed meaning-making, emotional regulation, and preparation for booster doses. The patient remained under medical supervision with routine vital-sign assessments until discharge on Day 13. While initial intake psychometrics confirmed severe substance use (DUDIT 34, AUDIT-C 3, CAGE 4), his CIWA-Ar score was 12 on the first night; no benzodiazepines or alcohol were administered. Baseline scores indicated moderate insomnia (ISI 15), alongside elevated depression and anxiety.

Over the next 3 to 5 days, the patient reported complete cessation of craving for ketamine, cocaine, alcohol, nicotine, cannabis, and caffeine. No arrhythmias, seizures, or psychosis occurred; QTc remained within normal limits. He was discharged on 20 November 2024, medically stable and craving-free. Three days later, he re-admitted to the German clinic for integration and monitoring. He remained abstinent and without withdrawal symptoms. Urine toxicology was repeatedly negative for all substances, though benzodiazepine metabolites remained detectable via confirmatory gas chromatography-mass spectrometry (GC/MS). These findings were interpreted as residual elimination of prior medication, consistent with long-acting benzodiazepine detection windows. Ethyl glucuronide remained <100 ng/mL. Psychometrically, his BDI-II score dropped to 14 (mild) and SCL-90-R indices normalized. Craving remained absent. After participating in daily psychotherapy and relapse prevention, he was discharged on 6 December 2024, clinically stable and abstinent. Two post-treatment 12-lead ECGs performed during the post-ibogaine integration stay at the German clinic confirmed normalization of cardiac repolarization: an ECG on 24 November 2024 (four days after discharge from the ibogaine center) showed sinus rhythm, HR 67/min, PQ 176 ms, normal R-progression, and QT/QTc within reference range; a second ECG on 5 December 2024 (the day before discharge from the integration stay) showed sinus rhythm, HR 82/min, normal R-progression, and QT/QTc within reference range. These findings, together with a pre-treatment 12-lead ECG on 31 October 2024 at the same German clinic (sinus rhythm 70/min, indifferent axis, normal QT/QTc), document a complete return to baseline cardiac repolarization following ibogaine exposure and bracket the index treatment with normal ECGs at both ends.

Outcome surveys were completed at multiple time points post-discharge. From January to May 2025, the Brief Substance Craving Scale and DSM-5 Level 2 Substance Use scores consistently remained 0. Depression (PHQ-9) decreased to 0–3 (minimal), and anxiety (GAD-7) improved to 0–4 (minimal/mild). PTSD symptom severity was assessed using the PTSD Checklist for DSM-5 (PCL-5; 26), a 20-item self-report measure of past-month DSM-5 PTSD symptoms with each item scored 0–4 and a total raw-score range of 0–80; the PCL-5 was administered via the OutcomeMD patient-reported-outcomes platform. OutcomeMD additionally reports a normalized score on a 0–100 scale on which higher values correspond to better outcomes (lower symptom burden); both the raw 0–80 score and the normalized 0–100 score are reported below to facilitate interpretation against the field-standard PCL-5 frame and the platform’s own visualization. PCL-5 scores decreased from a baseline raw score of 38 (OutcomeMD normalized 52.5) on the day of admission, above the commonly used clinical cutoff of 33 for probable PTSD, to 5 (normalized 93.75) by post-detoxification Day 8 immediately following ibogaine administration, with sustained reductions at one-month (raw 17/normalized 78.75) and four-month (raw 8/normalized 90) follow-up. The trajectory is consistent with clinically meaningful and durable improvement in trauma-related symptoms. Quality of life (WHOQOL-BREF) improved from ~55 pre-treatment to 71, with overall health satisfaction increasing from “neither satisfied nor dissatisfied” to “very satisfied.”

### Follow-up fractionated ibogaine intervention

In October 2025, he briefly re-engaged with the Mexico center for follow-up coaching; MEQ and CEQ assessments confirmed continued emotional processing without craving or relapse. Eleven months post-initial treatment, the patient returned following significant bereavement. Ibogaine was administered via a fractionated protocol over three sessions (500 mg, 300 mg, and 300 mg; 6.25, 3.75, and 3.75 mg/kg at a body weight of 80 kg; see **Table 2** for the full schedule). Transient QTc-prolongation occurred, but maximal values remained below 500 ms without arrhythmias or hemodynamic compromise. Mild bradycardia and ataxia resolved without intervention. Serial ECGs confirmed a return to baseline QTc-ranges, and the intervention was completed without medical complications.

**Table 2:** Ibogaine dosing schedule across the index treatment.

Visit	Date	Day	Session type	Weight (kg)	Dose (mg)	mg/ kg	Baseline QT/QTc (ms)	Peak QTc (ms)	Time to QTc <500 ms	IV electrolytes given	Adverse findings
1	14 Nov 2024	8	Flood dose	79	800	10.1	372/395 (pre-dose)	541 (~4 h post- dose)	~3h (below 500 by 15:00)	Mg 6 g. KCI 20 mEq	Transient ataxia, transient hypertension, emesis controlled with dimenhydrinate; no arrhythmia, no syncope, no hemodynamic instability
1	16 Nov 2024	10	Supplementary #1	79	300	3.8	404/417 (pre-dose)	454 (at 10:30)	n/a (peak <500)	Mg 2g. KCI 20 mEq	None of clinical significance
1	18 Nov 2024	12	Supplementary #2	79	300	3.8	384/402 (pre-dose)	444 (at 10:45)	n/a (peak <500)	Mg 2 g	None of clinical significance
2	5 Oct 2025	3	Fractionated #1	80	500	6.25	408/406 (pre-dose)	493 (at 11:30)	n/a (peak <500)	Mg 6g, KCI 40 mEq	Transient bradycardia, ataxia; no arrhythmia, no syncope
2	7 Oct 2025	5	Fractionated #2	80	300	3.75	436/441 (pre-dose)	478 (at 11:30)	n/a (peak <500)	Mg 4 g. KCI 20 mEq	Transient bradycardia (not below 40 bpm). ataxia; no arrhythmia
2	9 Oct 2025	7	Fractionated #3	80	300	3.75	388/396 (pre-dose)	452 (at 15:30)	n/a (peak <500)	Mg 2g	None of clinical significance

Doses, weight, mg/kg conversions, day-of-protocol, QTc time-course, IV electrolyte prophylaxis, and key adverse-event findings are reported as charted in the Beond nursing flow sheets. Visit 2 QTc values were calculated using the Fridericia correction (QTcF); the Visit 1 QTc correction formula was not specified in the available records.

At the last follow-up in April 2026, the patient remained abstinent from all previously misused substances. He reported a persistent absence of craving, stable mood with only mild intermittent symptoms, and functional reintegration into work and relationships. As of April 2, 2026, he remains abstinent.

### Psychological and functional outcomes following ibogaine treatment

Analysis of psychological and functional outcomes revealed improvements across key metrics. The Insomnia Severity Index (ISI) improved from 15 (Moderate Insomnia) pre-treatment, though mild insomnia persisted. Existential functioning (MLQ) remained stable at 52, indicating strong coherence. Baseline resilience (BRS) was 12 (low-to-moderate), though this was not repeated in follow-up. Finally, the Rosenberg Self-Esteem (RSE) score of 15 showed clinical improvement, indicating self-regard was under reconstruction following the intervention.

### Patient´s perspective

“Reflecting on my recovery, I believe the synergy between conventional therapy and the psychedelic-assisted sessions was the catalyst for change. However, it was the intentional period of institutional integration that proved most vital; having the time to process these insights without the immediate pressure of returning to ‘normal’ life ensured that I was truly ready for the world, and that my life was restructured to support my new self. “

## Discussion

This case provides the first rigorously documented evidence of sustained remission from severe, treatment-resistant ketamine dependence following ibogaine therapy. While ibogaine’s efficacy in opioid and cocaine use disorders is increasingly recognized, its application in complex polysubstance cases, particularly those involving chronic ketamine misuse, remains largely unexplored. The complete cessation of craving over approximately 17 months, validated here by longitudinal psychometrics and toxicology, represents a clinical outcome that significantly exceeds the prognostic expectations for this high-risk population in conventional psychiatric settings.

The simultaneous cessation of craving across multiple substance classes, ketamine, cocaine, and alcohol, supports the hypothesized multi-target mechanism of iboga alkaloids. The neurobiological mechanisms discussed throughout this report, however, should be interpreted as plausible hypotheses informed by preclinical and clinical literature rather than conclusions that can be established from a single clinical observation. We propose a biphasic recovery process: the acute interruption of compulsive use is likely driven by ibogaine’s NMDA-antagonism and α3β4 nAChR modulation, which disrupt the dopamine spikes and cue-conditioned reinforcement essential to both cocaine and ketamine dependence (11, 23). The sustained 17-month remission is further attributed to the long-acting metabolite noribogaine (**Table 3**). Its KOR-agonism may underpin rapid anti-dysphoric effects, while its SERT-inhibition correlates with the stabilization of depressive and anxiety symptoms observed in this patient’s serial PHQ-9 and BDI-II scores (24). This pharmacological synergy likely initiates a metaplastic state, creating a therapeutic window for the long-term functional reintegration discussed below (4).

**Table 3:** Longitudinal psychometric and clinical outcomes.

A: Mood, anxiety, and trauma				
Timepoint	Setting	Depression (BDI-II/PHQ-9)	Anxiety (GAD-7)	PTSD (PCL-5)
Pre-Ibogaine (30 Oct 2024)	German Clinic	BDI-II: 32 (Severe)	Not recorded	Not recorded
Immediately Pre- Ibogaine (7 Nov 2024)	Beond Mexico	PHQ-9: ≈18–20 (Moderately severe)	GAD-7: Moderate– Severe (≈15+)	Documented trauma exposure, no PCL-5
Post-Ibogaine (23 Nov 2024)	German Clinic	BDI-II: 14 (Mild)	Anxiety: low-moderate	Not measured
Early Follow-up (Jan 2025)	Beond Mexico	PHQ-9: 0–2 (Minimal)	GAD-7: 0–3 (Minimal)	PCL-5: ~55 (Improving from ~90 pre- treatment)
Mid Follow-up (May 2025)	Beond Mexico	PHQ-9: 0–3	GAD-7: 0–4	PCL-5: ~52–55
Latest (April 2026)	Beond Mexico	PHQ-9: 1–4	GAD-7: 3	No PTSD symptoms causing impairment
B. Craving and Substance Use				
Timepoint	Instrument	Craving score	Substance use: notes	
Pre-Ibogaine (7 Nov 2024)	DUDIT	34 (Severe)	Ketamine daily, cocaine weekly, alcohol frequent High dependence	
	CAGE	4 (Positive for alcoholism)		
	Substance Craving (self-report)	High	Active use	
Post-Ibogaine (23 Nov 2024)	Clinical Observation	0 No Craving	Abstinent (confirmed by urine tox); Patient reports cessation of desire for all substances (including caffeine + nicotine)	
Jan-25	Brief Substance Craving Scale	0	None in past 7 days: Frequency: Never	
May-25	Brief Substance Craving Scale	0	None	
Apr-26	DSM-5 Level 2 — Substance Use	0	None: Sustained remission	
C. Functional Status and Quality of Life				
Timepoint	Parameter	Score	Interpretation	
07 Nov 2024	WHOQOL-BREF Total	55/100	Low QoL	
	Health Satisfaction	3 (Neutral)	Health neither good nor poor	
17 Apr 2026	WHOQOL-BREF Total	71/100	Moderate–High QoL	
	Health Satisfaction	5 (Very satisfied)	Significant improvement	
	Daily functioning	Improved	From “dissatisfied” → “satisfied”	
	Work ability	3 → 5 (neutral → very satisfied)		
	Enjoyment of life	3 → 4	Moderate → high enjoyment	
	Sense of meaning	Stable high (4→4)	High meaning maintained	

Longitudinal Psychometric and Clinical Outcomes for the Patient Pre-Ibogaine, Post-Ibogaine, and through 17-Month Follow-up (April 2026). The data are presented in a stacked format to reflect changes across Mood, Craving, and Functional Status.

### The ketamine paradox

Chronic ketamine dependence presents a pharmacological paradox: both substances interact with NMDA receptors, yet they shape neural circuitry in opposing directions. Repeated ketamine misuse has been associated with maladaptive neuroplastic changes affecting glutamate homeostasis, receptor-level adaptations, and dysfunction within prefrontal-limbic circuits implicated in reward processing and behavioral control. Specifically, chronic ketamine exposure produces compensatory upregulation of NMDA receptor subunits and dysregulated metabotropic glutamate receptor 5 (mGluR5) signaling, which together amplify cue-evoked excitability in the medial prefrontal cortex and disrupt prefrontal-to-nucleus accumbens top-down control of reward-seeking behavior (27, 28). Concurrent elevations in serum BDNF (2) and dissociation-associated alterations in cortical glutamate–GABA balance are thought to consolidate these adaptations into compulsive use patterns (3). In contrast, ibogaine and noribogaine engage multiple neurotransmitter systems simultaneously — serotonergic, dopaminergic, opioid, and cholinergic — and the observed clinical improvement is therefore more plausibly understood as a broader network-level reorganization than as a direct reversal of ketamine’s NMDA-mediated effects (10, 11). Critically, the metabolite noribogaine has negligible NMDA-binding, suggesting that the sustained therapeutic effects in this case were mediated by non-NMDA targets (10). As a KOR-agonist and SERT-inhibitor, noribogaine likely counteracted the neuroadaptive changes, such as downregulated dopaminergic tone and altered reward processing, typically seen in long-term ketamine misuse (29). Furthermore, while ketamine-induced neuroplasticity is often transient and dissociative, ibogaine’s induction of GDNF and BDNF expression in mesolimbic circuits (30) supports a more stable, long-term reorganization of the reward system. This reorganization of the reward system is likely underpinned by the potent upregulation of BDNF and GDNF via TrkB-activation, which may effectively re-open critical periods and facilitate the extinction of drug-seeking behaviors (15, 30). These mechanisms, considered together, address the neurobiological components of chronic ketamine dependence; however, as noted above, they remain hypothetical in the context of a single clinical observation and require validation in controlled studies.

### Ketamine metabolites and opioid-receptor modulation

The question of whether the clinical picture presented here could reflect, in part, an opioid-related disorder rather than a pure dissociative-use phenotype deserves explicit consideration. Chronic ketamine use produces multiple long-acting metabolites, most notably hydroxynorketamine (HNK), that exhibit positive allosteric modulation at μ-opioid receptors (29, 31). Williams et al. (31) demonstrated that the rapid antidepressant effects of ketamine in humans are attenuated by the opioid-receptor antagonist naltrexone, providing direct clinical evidence that opioid-receptor signaling contributes to at least part of ketamine’s central effects. Bonaventura et al. (29) further documented enantiomer-specific divergence in ketamine’s opioid-related pharmacology, with implications for drug-abuse liability. Whether chronic, high-dose ketamine misuse, as in the present case, produces a clinical phenotype that overlaps mechanistically with opioid use disorder remains an open question. Current DSM-5 nosology classifies ketamine use disorder under hallucinogen/dissociative-related disorders rather than opioid-related disorders, but the neurobiological substrate is likely multifactorial, involving glutamatergic, dopaminergic, serotonergic, and opioid-receptor-related mechanisms. From a treatment-mechanism standpoint, ibogaine’s noribogaine-mediated κ-opioid agonism (10) may engage opioid-system pathways relevant to this multi-pathway picture, providing a parsimonious, though hypothetical, account of why a substance with mixed opioid-modulating activity might disrupt craving in a patient whose substance was not classified as an opioid. We emphasize that this remains a hypothesis to be tested in controlled studies.

### Pharmacokinetic stabilization

A central hypothesis for this patient’s success is that pharmacokinetic timescales dictate craving dynamics. Ketamine is notably short-acting (t1/2 ≈ 2–4h), which, when used compulsively, promotes frequent re-dosing and rapid oscillations between intoxication and withdrawal-induced craving (32). In contrast, ibogaine produces a significantly longer therapeutic tail via its active metabolite, noribogaine, which is eliminated slowly (t1/2 ≈ 28–49h) (8). This prolonged exposure likely smoothed the peaks and troughs that typically reinforce compulsive use, providing a stable physiological baseline during the critical early weeks of abstinence. By reducing acute cue-reactivity during this window, the extended half-life of noribogaine allowed for the successful extinction of drug-seeking habits that usually defeat short-term detoxifications.

### Future directions & experiential mediators

The therapeutic potential of iboga alkaloids is further illuminated by recent advancements in rational drug design. Work on oxa-iboga analogs suggests that anti-addictive properties, primarily mediated by SERT, DAT, and KOR modulation, are pharmacologically separable from cardiac liabilities such as hERG-binding and QTc prolongation (33). Interestingly, because these analogs retain efficacy despite reduced 5-HT2A activity, classical psychedelic signaling may not be the primary driver of the anti-addictive response. However, the psychological dimension remains significant; recent clinical evidence suggests that ibogaine can elicit mystical-type experiences (16, 34). In this case, such experiences may have served as a meaningful mediator of change, potentially reducing PTSD-related symptom severity and providing the existential shift necessary to sustain long-term abstinence. This experiential shift likely coincided with the reopening of an oxytocin-dependent critical period for social-emotional learning (15). By temporarily returning the brain to a state of heightened metaplasticity, ibogaine may have provided a unique window for the patient to unlearn compulsive drug-seeking habits and re-establish the social and emotional regulation necessary for approximately 17 months of successful recovery.

### Limitations

This case study’s longitudinal outcome is subject to limitations inherent to single-subject designs, which restrict generalizability. Observed effects cannot be definitively attributed solely to ibogaine, given concurrent integration therapy, nutritional stabilization, and the absence of any pharmacological control condition (10, 21). The non-regulated international setting required discontinuing formal psychiatric care in Germany, presenting ethical and safety concerns regarding continuity. The fractionated ibogaine intervention administered approximately eleven months after initial treatment following a significant bereavement event represents a substantial confounding variable when interpreting long-term outcomes. Subsequent changes in PTSD symptoms, mood, quality of life, and sustained abstinence may have been influenced by a combination of grief processing, psychosocial adaptation, and additional therapeutic exposure rather than the effects of the original ibogaine intervention alone; the PTSD trajectory may reflect grief-processing rather than a specific ibogaine effect. Finally, reliance on self-report and remote monitoring, despite toxicology confirmation, introduces potential reporting bias common in observational follow-up.

### Ethical challenges in cross-border psychedelic care

This case highlights emerging ethical challenges associated with cross-border psychedelic treatment. The patient voluntarily left a regulated psychiatric inpatient program in Germany to pursue ibogaine treatment in a jurisdiction where such care was legally available but not subject to the same regulatory framework. This raises important questions regarding informed refusal, patient autonomy, continuity of care, and the responsibilities of clinicians when patients seek interventions unavailable within their home healthcare systems. Upon the patient’s return, the treating team was required to balance awareness of ibogaine’s recognized risks with an ongoing duty of care toward an individual reporting substantial clinical benefit. As international travel for psychedelic treatment becomes increasingly common, clearer ethical and professional guidance may be needed regarding post-treatment monitoring, reintegration support, and management of potential complications.

### Ethical and safety considerations

The use of ibogaine introduces significant ethical and safety concerns, primarily related to its potential for cardiotoxicity (QTc prolongation and arrhythmia risk). Ethically, this case highlights the conflict posed by a patient seeking an unapproved intervention in an unregulated international facility, necessitating the German psychiatric clinic’s subsequent role in integrating a therapy that carries known mortality risks. This practice underscores the need for formalized ethical guidelines and robust safety protocols in licensed settings before widespread adoption.

Regarding the cardiac-safety findings observed in this case, a QTc peak of 541 ms during the flood-dose session warrants explicit discussion against current safety thresholds. Published consensus guidance treats QTc values >500 ms as a marker of substantially elevated torsades-de-pointes risk warranting close monitoring or therapeutic intervention; values in this range during ibogaine exposure have historically been associated with the few documented serious cardiac events in the literature (18, 21). In the present case, however, the prolongation was strictly transient, peaking at Hour 4 (~4 h post-administration), resolving below 500 ms by Hour 7 under prophylactic IV magnesium (total 6 g) and potassium chloride (total 20 mEq) supplementation, and not associated with any arrhythmia, torsades de pointes, syncope, or hemodynamic instability. No antiarrhythmic intervention was required. Two ECGs performed two days and three weeks after discharge confirmed complete normalization of cardiac repolarization. This time course is consistent with the dose-related, reversible QTc-prolonging profile of noribogaine documented in the ascending-dose pharmacokinetic study by Glue et al. (7), in which QTc returned to baseline as serum noribogaine concentrations declined. While these findings do not establish 541 ms as a clinically safe threshold, published safety guidance continues to treat such values as warranting active risk management, they suggest that, under continuous 12-lead ECG monitoring, electrolyte optimization, and prophylactic IV magnesium/potassium supplementation, transient QTc excursions of this magnitude can resolve without adverse clinical sequelae in appropriately selected patients. For future cases, this argues for rigorous pre-treatment risk stratification: 12-lead ECG screening with calculation of QTc using a validated correction formula (Fridericia or Hodges rather than Bazett at the heart rates observed during dosing), a personal and family history of long-QT syndrome, screening for QT-prolonging concomitant medications (e.g., methadone, certain SSRIs, antipsychotics, fluoroquinolones, ondansetron), serum electrolyte optimization (potassium, magnesium, calcium) prior to dosing, and intra-procedural continuous ECG monitoring with the immediate availability of antiarrhythmic capability. Patients with baseline QTc >450 ms, structural heart disease, or unmodifiable QT-prolonging medications should be approached with substantially heightened caution.

The two supplementary-dose sessions showed substantially smaller QTc excursions than the flood dose (Day 10: 417→454 ms; Day 12: 402→444 ms; see **Table 1**), each conducted under continuous cardiac monitoring with serial ECG and intravenous magnesium supplementation, and neither was associated with ventricular arrhythmia, torsades de pointes, syncope, or hemodynamic instability.

## Data Availability

The raw data supporting the conclusions of this article will be made available by the authors, without undue reservation.
