# Correction: Sustained abstinence in severe ketamine use disorder following ibogaine treatment case report

**DOI:** 10.3389/fpsyt.2026.2007932

**Published:** 2026-09-30

**Authors:** Sergio R. Pérez Rosal, Sonya C. Faber, Markus Backmund

**Affiliations:** 1 Medizinische Hochschule Brandenburg Theodor Fontane, Neuruppin, Germany; 2 Psychedelia-Stiftung, Berlin, Germany; 3 School of Psychology, University of Ottawa, Ottawa, ON, Canada; 4 P3 Akutklinik für Psychiatrie, Psychotherapie und Psychosomatik, Tutzing, Germany; 5 Ludwig-Maximilians-Universität (LMU) Munich, Munich, Germany

**Keywords:** case report, ibogaine, ketamine use disorder (KUD), psychoactive substances, substance use disorder (SUD)

Author Sonya C. Faber was erroneously omitted as equal contributing author. The original version of this article has been updated.

The original version of this article has been updated.

