# Supplementary material for: Sustained abstinence in severe ketamine use disorder following ibogaine treatment case report
Source: Front Psychiatry. 2026 Jul 20;17:1846320. doi: 10.3389/fpsyt.2026.1846320 (PMC13430120; doi:10.3389/fpsyt.2026.1846320)
Supplement: Supplementary file 1 [file Table1.docx]

**Supplementary Table: Summary of Clinical Evidence and Dose**

| **Study type** | N | Compound | Population | Key outcomes | Safety notes |
| --- | --- | --- | --- | --- | --- |
| Retrospective survey | 75 | Ibogaine | SUD (mixed) | ↓ withdrawal; median abstinence 5–8 mo | – |
| Retrospective survey | 88 | Ibogaine | OUD | ↓ withdrawal (80%); ↓ craving up to 3 mo | – |
| Observational | 51 | Ibogaine → 5-MeO-DMT | Veterans, PTSD | ↓ PTSD, depression, anxiety | – |
| Observational | - | Ibogaine | OUD | ↓ withdrawal | Fatality; poor monitoring |
| Phase I | 21 | Ibogaine (20 mg) | Healthy adults | No cognitive effect | Safe |
| Phase I | 9 | Noribogaine (5 days) | OUD on OAT | ↓ withdrawal; ↑ mood | Dropouts |
| Open-label | 32 | Ibogaine HCl | OUD | ↓ withdrawal | No SAE |
| Open-label | 191 | Ibogaine HCl | OUD/CUD | ↓ craving; ↓ depression; ↓ mood symptoms | No SAE |
| RCT (DBPCT) | 20 | Ibogaine | CUD | ↓ craving; fewer relapses | – |
| RDBPCT | - | Noribogaine | OUD | ↓ withdrawal (NS); QTc ↑ dose-dependent | No SAE |
|  | | | | | |
| **Dose category** | Approx range | | Context / notes | | Safety signals |
| Proposed safe initial dose | ~0.87 mg/kg | | Suggested safe threshold | | No major concerns |
| Lowest reported dose | ~0.28 mg/kg | | Glue et al. 2015 | | Well tolerated |
| Common clinical dosing | ~5–25 mg/kg | | Case series, observational, open-label | | Reduced withdrawal; QTc possible |
| High-dose use | Up to 55 mg/kg | | Noller et al. 2018 | | Associated fatality |
| Microdosing | Repeated low dosing | | Anecdotal reports | | Potential benefit; minimal data |

adapted from information in Köck et al., 2022
